# Geriatric nutritional risk index predicts poor outcomes in patients with acute ischemic stroke - Automated undernutrition screen tool

**DOI:** 10.1371/journal.pone.0228738

**Published:** 2020-02-13

**Authors:** Min Kyoung Kang, Tae Jung Kim, Yerim Kim, Ki-Woong Nam, Han-Yeong Jeong, Sung Kyung Kim, Ji Sung Lee, Sang-Bae Ko, Byung-Woo Yoon

**Affiliations:** 1 Department of Neurology, Seoul National University Hospital, Seoul, Republic of Korea; 2 Department of Neurology, Seoul National University College of Medicine, Seoul, Republic of Korea; 3 Department of Neurology, Kangdong Sacred Heart Hospital, Hallym University Medical Center, Seoul, Republic of Korea; 4 Medical Research Collaborating Center, Seoul National University Hospital, Seoul, Republic of Korea; 5 Clinical Research Center, Asan Medical Center, Seoul, Republic of Korea; University of Ioannina School of Medicine, GREECE

## Abstract

**Background:**

Premorbid undernutrition has been proven to have an adverse effect on the prognosis of stroke patients. The evaluation of nutritional status is important, but there is no universally accepted screen methodology.

**Purpose:**

We aimed to use the geriatric nutritional risk index (GNRI) for evaluating the effect of premorbid undernutrition on short-term outcomes in patients with acute ischemic stroke.

**Methods:**

A total of 1,906 patients were included for analysis. Baseline characteristics were collected. We evaluated the nutritional status of the patients using the GNRI and body mass index(BMI). The GNRI was calculated as {1.519×serum albumin(g/dL) + 41.7×present weight (kg)/ideal body weight (kg)}. All patients were categorized into four groups on the basis of the GNRI score.

**Results:**

Among the included patients, 546 patients had an unfavorable outcomes. The proportion of patients with moderate and severe risk, assessed in GNRI, was significantly higher in the unfavorable outcome group compared to the favorable outcome group (33.3% vs 15.0%). The increased risk of premorbid undernutrition was associated with an increased risk of unfavorable outcome in a dose-response manner after adjusting for covariates.

**Conclusions:**

This study demonstrated that GNRI was associated with poor prognosis in patients with acute ischemic stroke. GNRI may be used to screen patients at high risk for unfavorable outcome.

## Introduction

Undernutrition is defined as a long-standing negative imbalance in intake and requirement of both energy and protein. Premorbid undernutrition has been proven to have an adverse effect on the prognosis of stroke patients [1–2]. It is associated with increases in complications, mortality, length of hospitalization, and poor neurological outcomes in acute stroke patients [3–4]. Therefore, it is important to evaluate nutritional status appropriately and provide proper nutritional supplement. However, there is currently no universally accepted screen methodology for nutritional assessment. Several nutritional assessment tools are based on anthropometry, morbidity, subjective evaluation of patients using a questionnaire [5–6]. However, there are practical difficulties in assessing the nutritional status of all patients with acute stroke using these tools. The geriatric nutritional risk index (GNRI) is known as an objective measurement of nutrition based on biochemical and body indexes in patients with malignancy and cardiovascular disease [7–8]. It is a simple tool using objective information, and does not require a nutritional specialist or the patient’s cooperation.

In this study, we aimed to explore the association between nutritional status assessed early after an acute ischemic stroke using the GNRI and short-term outcomes following acute ischemic stroke.

## Methods

### Study population

From January 2010 and December 2016, we screened 2,084 patients with acute ischemic stroke who were admitted within seven days of symptom onset based on the single center prospective registry system (since October 2002). We excluded patients with the following conditions: lack of laboratory information or dysphagia test within 24 hours of admission (n = 72), no modified 3-month Rankin Scale (mRS) score data after hospitalization (n = 106). Because early dysphagia assessment could be a concomitant factor for prevention of complication and prognosis of ischemic stroke, the patients with absence of dysphagia assessment within 24 hours were excluded [9]. Finally, a total of 1,906 patients were included for analysis. The institutional review board of Seoul National University Hospital approved the study protocol and waived the need for patient consent (IRB NO. 1009-062-332).

### Clinical information

Baseline characteristics, including age, gender, body mass index (BMI), premorbid mRS score data, history of hypertension, diabetes mellitus (DM), dyslipidemia, current smoking (last cigarette within 6 months), previous stroke/transient ischemic stroke (TIA), and heart disease such as atrial fibrillation or coronary heart disease, were collected. In addition, chronic conditions related to malnutrition such as gastrointestinal disease (mechanical obstruction, intestinal fistula, inflammatory bowel disease), chronic obstructive pulmonary disease, chronic renal disease, and malignancy treated within the past 6 months (esophagus, stomach, colon, rectum, liver, pancreas, lung, head and neck cancer, leukemia, lymphoma or sarcoma) were evaluated [10–14]. The height and weight of the patient on admission were measured using an automatic scale (Model GL-150, G-Tech International, Uijeongbu-si, Gyeonggi-do, South Korea) by skilled nurses. In cases of severe stroke patients who could not stand alone, we measured the body weight using an underbed scale and height using a tapeline [2]. Laboratory information on leukocyte count, hemoglobin, serum albumin, serum total protein, low-density lipoprotein (LDL) cholesterol, hemoglobin A1C, serum creatinine and C-reactive protein (CRP) was collected from the electronic medical record. For evaluating the initial neurological severity, the National Institutes of Health Stroke Scale (NIHSS) score was assessed on admission. We classified the stroke subtypes according to the Trial of Org 10172 in Acute Stroke Treatment (TOAST) [15].

### Evaluating nutritional status

We evaluated nutritional status early after stroke using the GNRI. The GNRI was calculated as {1.519×serum albumin(g/dL) + 41.7×present weight(kg)/ideal body weight(kg)} [16]. The ideal body weight (IBW) was calculated according to the Lorentz formula calibrated for the patient’s height and sex as follows:

"formen:IBW=height(cm)‐100‐{(height(cm)‐150)/4}"

"forwomen:IBW=height(cm)‐100‐{(height(cm)‐150)/2}"

All patients were categorized into four groups on the basis of their GNRI score: 1) severe risk (GNRI<82); 2) moderate risk (82≤GNRI<92); 3) mild risk (92≤GNRI<98); and 4) no risk (GNRI≥98) [17].

### Outcome measures

We evaluated the short-term outcomes using a 3-month mRS score after stroke onset via an outpatient visit or structured telephone interview. We divided patients into two groups with favorable outcome (mRS score≤2) and unfavorable outcome (mRS score≥3) [18]. We compared the clinical characteristics, laboratory data, and premorbid undernutrition risk evaluated by the GNRI score between the two groups. For the comparison of nutritional status, the patients were divided into four groups by their GNRI score as described above.

### The comparison of two nutritional screening methods on outcomes

The receiver operating characteristic (ROC) analysis was conducted by plotting the sensitivity against the value of 1-specificity for assessing the performance of the GNRI and BMI on predicting unfavorable short-term outcome after acute ischemic stroke. Areas under the ROC curve (AUC) were compared to examine how well nutritional screening methods predicted clinical outcome.

### Statistical analysis of the clinical data

**Fig 1:**
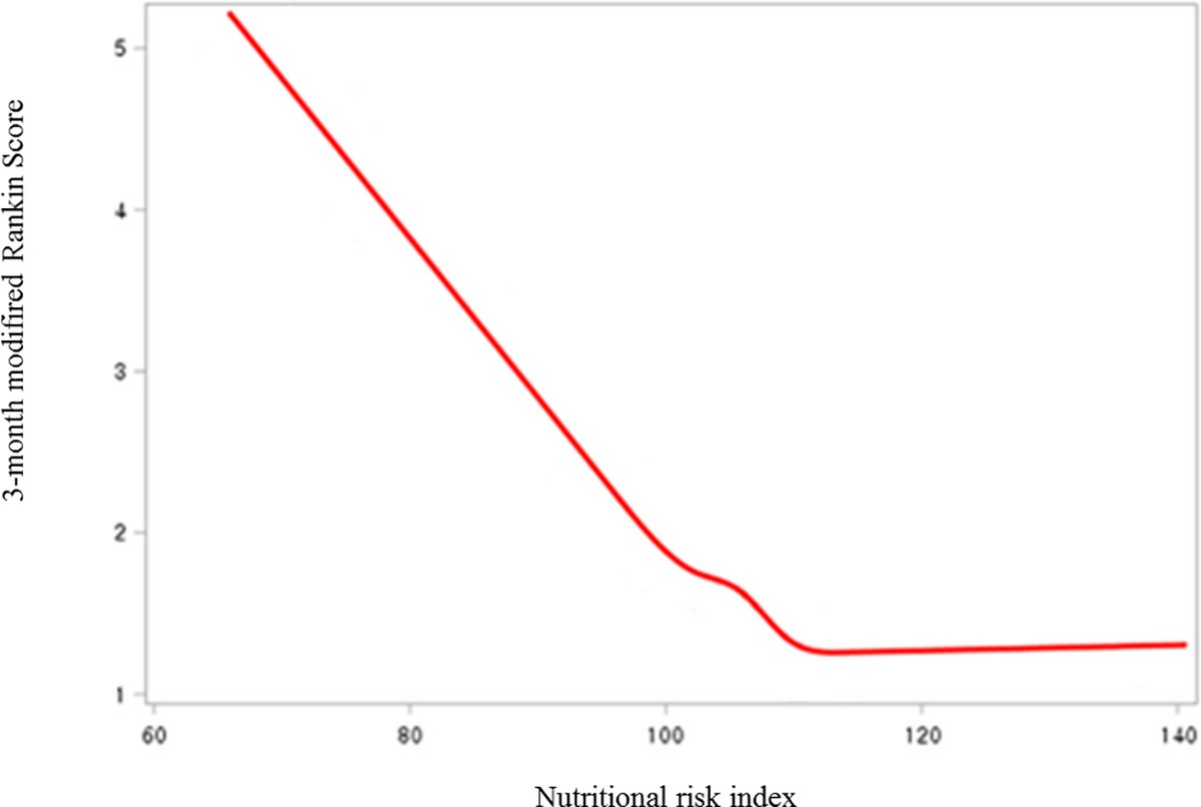
Distribution of nutritional status assessed by geriatric nutritional risk index and percentage of modified Rankin Score at 3 months after ischemic stroke cases (red line). The restricted cubic spline model was used to determine the distribution of the data.

Analyses were performed using the SPSS program (Version 23.0, IBM Statistics) and SAS 9.4 software (SAS Studio 3.7, SAS institute). Graphics and comparison of ROC analyses were performed using SAS 9.4 software (SAS Studio 3.7, SAS institute). The distribution of clinical characteristics, laboratory data, and stroke subtype data were compared using a Student’s t-tests for continuous variables, Pearson’s χ^2^ tests for categorical variable, one-way analysis of variance with post-hoc Duncan’s test for the four nutritional risk status groups, and Fisher’s exact test, the Mann-Whitney U-test, and the Kruskal-Wallis H test for nonparametric variables. We analyzed the relationship between the GNRI value and outcome using the restricted cubic spline function, and GNRI value of 100 was chosen as the reference value (Fig 1). Covariates with P<0.05 in the univariate analysis and those with clinically important factors were adjusted for multivariate analysis.

## Results

### Clinical characteristics of the patients with unfavorable outcome

Among the included patients, the mean age was 67 years and 1,168 (59.8%) patients were male (Table 1). Of the 1,906 patients, 546 (28.6%) had an unfavorable outcome. The unfavorable outcome group, in the univariate analysis, was more likely to be older, female and more likely to have a history of hypertension, diabetes mellitus, atrial fibrillation, and a previous history of stroke or transient ischemia attack (TIA). The stroke mechanisms were more likely to be cardioembolic or other determined causes. Premorbid mRS score, initial NIHSS scores and discharge NIHSS scores were higher in the unfavorable outcome group. The unfavorable outcome group had lower hemoglobin, total protein, serum albumin, and LDL cholesterol level. They also had a higher leukocyte count, and CRP level. The proportion of patients with moderate and severe risk was significantly higher in the unfavorable outcome group than in the favorable outcome group. In addition, the unfavorable outcome group had a lower BMI and serum albumin level. The mean duration from admission to nutritional assessment was 0.4±0.2 day, with no difference between the two groups.

**Table 1:** Baseline characteristics according to 3-month outcomes.

Variables	Favorable outcome (N = 1360, 71.4%)	Unfavorable outcome (N = 546, 28.6%)	P-value
Age, mean(SD*), year	66.19 ± 12.07	71.69 ± 12.01	<0.001
**Male, n(%)**	881(64.8)	287(52.6)	<0.001
Weight, median(IQR*), Kg	63.6 [56.0–70.5]	58.7 [51.5–65.5]	<0.001
BMI*, median(IQR*), Kg/m^**2**^	23.6 [21.7–25.6]	22.5 [20.2–25.0]	0.001
**Hypertension, n(%)**	838(61.6)	373 (68.3)	0.006
**Diabetes Mellitus, n(%)**	406(29.9)	208(38.1)	<0.001
**Dyslipidemia, n(%)**	520(38.2)	179(32.8)	0.027
Previous stroke/TIA*, n(%)	246(18.1)	156(28.6)	<0.001
**Atrial fibrillation, n(%)**	242(17.8)	165(30.2)	<0.001
**Smoking, n(%)**	364(26.8)	154(28.2)	0.072
**Coronary heart disease, n(%)**	156(11.5)	64(11.7)	0.874
Premorbid mRS*, median(IQR*)	0 [0–0]	0 [0–2]	<0.001
Initial NIHSS*, median(IQR*)	1 [1–5]	9 [4–15]	<0.001
Discharge NIHSS*, median(IQR*)	1 [0–2]	6 [2–12]	<0.001
**Stroke, mechanism, n(%)**			<0.001
LAA*	443(32.6)	163(29.9)	
SVO*	297(21.8)	68(12.5)	
CE*	318(23.4)	175(32.1)	
**Other determined**	97(7.1)	74(13.6)	
**Undetermined**	205(15.1)	66(12.1)	
**Nutritional risk index, n(%)**			<0.001
**No risk**	1077(79.2)	321(58.8)	
**Mild risk**	79(5.8)	43(7.9)	
**Moderate risk**	193(14.2)	147(26.9)	
**Severe risk**	11(0.8)	35(6.4)	
**Laboratory parameters**			
Leukocyte count, mean(SD*), 10^**3**^**/uL**	7.95 ± 2.65	8.61 ± 3.37	<0.001
Hemoglobin, mean(SD*), g/dL	13.71 ± 1.87	12.90 ± 2.21	<0.001
Albumin, mean(SD*), g/dL	4.09 ± 0.38	3.85 ± 0.49	<0.001
Total protein, mean(SD*), g/dL	7.05 ± 0.56	6.90 ± 0.68	<0.001
LDL* cholesterol, mean(SD*), mg/dL	106.17 ± 40.71	99.15 ± 46.00	0.001
Creatinine, mean(SD*), mg/dL	1.08 ± 1.06	1.10 ± 0.99	0.732
C-reactive protein, mean(SD*), mg/dL	0.65 ± 2.23	2.02 ± 4.42	<0.001

*Abbreviation: SD, standard deviation; IQR, interquartile range; BMI, body mass index; TIA, transient ischemia attack; mRS, modified rankin scale; NIHSS, National Institutes of Health Stroke Scale; LAA, large artery atherosclerosis; SVO, small vessel occlusion; CE, cardioembolism; LDL, low-density lipoprotein.

### The correlation between nutritional status and outcome

In univariate analysis, the younger patients tended to belong to no risk group, whereas older people were approximately equally distributed in the mild, moderate, and severe risk groups (Table 2). In contrast, patients with a history of atrial fibrillation tended to belong to mild, moderate, and severe risk groups. A history of hypertension or dyslipidemia was more prevalent in the no and mild risk groups. When comparing the baseline characteristics according to the GNRI score, the lower GNRI group (moderate and severe risk of premorbid undernutrition group) were more likely to have lower BMI and GNRI scores, more likely to have a history of chronic condition related to premorbid undernutrition, especially a history of malignancy. The patients with a lower GNRI group had more likely to be cardioembolic or other determined subtype stroke, significantly higher premorbid mRS score, initial and discharge NIHSS scores. The higher risk of premorbid undernutrition was associated with an increased risk of unfavorable outcome in a dose response manner after adjusting for age, sex, history of hypertension, DM, DL, previous stroke or TIA, atrial fibrillation, stroke subtype, previous mRS and initial NIHSS score (Moderate risk odds ratio (OR) 1.522; 95% confidence interval (CI) 1.110–2.086; P = 0.009: Severe risk OR 3.838; 95% CI 1.727–8.529; P<0.001, respectively; Table 3).

**Table 2:** Baseline characteristics according to the initial nutritional status.

Variables	No risk (N = 1398, 73.3%)		Mild risk (N = 122, 6.4%)	Moderate risk (N = 340, 17.9%)	Severe risk (N = 46, 2.4%)	P-value
Age, mean(SD*), year	66.2 ± 12.2		71.16 ± 11.52	72.32 ± 11.36	71.35 ± 13.24	<0.001
**Male, n(%)**	857(61.3)		77(63.1)	193(56.8)	33(71.7)	0.397
BMI*, median(IQR*), Kg/m^**2**^	24.3[22.6–26.1]		21.1[19.8–22.3]	20.3[19.0–22.2]	18.4[17.4–19.8]	<0.001
Geriatric Nutritional risk index, median(IQR*), unit	106.7[102.7–111.3]		96.8[96.2–97.5]	90.6[88.4–93.6]	77[73.8–79.7]	<0.001
**Hypertension, n(%)**	914(65.4)		83(68.0)	187(55.0)	27(58.7)	0.003
**Diabetes Mellitus, n(%)**	437(31.3)		41(33.6)	120(35.3)	16(34.8)	0.511
**Dyslipidemia, n(%)**	570(40.8)		39(32.0)	79(23.2)	11(23.9)	<0.001
Previous stroke/TIA*, n(%)	284(20.3)		26(21.3)	82(24.1)	10(21.7)	0.495
**Atrial fibrillation, n(%)**	271(19.4)		33(27.0)	93(27.4)	10(21.7)	0.005
**Smoking, n(%)**	378(27.0)		35(28.7)	87(25.6)	18(37.0)	0.074
**Coronary heart disease, n(%)**	157(11.2)		14(11.5)	45(13.2)	4(8.7)	0.693
Premorbid mRS*, median(IQR*)	0 [0–1]		0 [0–1]	0 [0–1]	0 [0–3]	<0.001
Initial NIHSS*, median(IQR*)	3[1–6]		4 [1–10]	5 [2–11]	9 [5–18]	<0.001
**Stroke severity, n(%)**						<0.001
Mild (NIHSS*≤8)	1168(83.5)		87(71.3)	221(65.0)	21(45.7)	
Moderate (9≤NIHSS*≤15)	145(10.4)		20(16.4)	69(20.3)	9(19.6)	
Severe (NIHSS*≥16)	85(6.1)		15(12.3)	50(14.7)	16(34.7)	
Discharge NIHSS*, median(IQR*)	3 [0–4]		2 [0–5]	3 [1–7]	5 [2–16]	<0.001
**Stroke, mechanism, n(%)**						<0.001
LAA*	467(33.4)		39(32.0)	96(28.2)	4(8.7)	
SVO*	306(21.9)		16(13.1)	39(11.5)	4(8.7)	
CE*	338(24.2)		37(30.3)	110(32.4)	8(17.4)	
**Other determined**	96(6.9)		10(8.2)	47(13.8)	18(39.1)	
**Undetermined**	191(13.7)		20(16.4)	48(14.1)	12(26.1)	
**Chronic condition related to malnutrition, n(%)**		165(11.8)	33(27.0)	69(20.3)	24(52.2)	<0.001
**Gastrointestinal disease**	46(3.3)		8(6.6)	15(4.4)	8(17.4)	
**Chronic obstructive pulmonary disease**	14(1.0)		0(0.0)	2(0.6)	1(4.2)	
**Chronic renal disease**	32(2.3)		2(1.6)	8(2.4)	4(8.7)	
**Malignancy**	73(5.2)		23(18.9)	44(12.9)	11(23.9)	
**Laboratory feature**						<0.001
Leukocyte count, mean(SD*), 10^**3**^**/uL**	8.01 ± 2.63		8.47± 3.42	8.30 ± 3.24	9.95 ± 4.68	
Hemoglobin, mean(SD*), g/dL	13.96±1.68		12.92±2.23	12.14 ± 2.05	10.19 ± 2.06	
Albumin, mean(SD*), g/dL	4.18 ± 0.29		3.88 ± 0.26	3.57± 0.34	2.82 ± 0.45	
Total protein, mean(SD*), mg/dL	7.18± 0.48		6.83 ± 0.57	6.54 ± 0.61	5.99 ±1.10	
LDL* cholesterol, mean(SD*), mg/dL	107.98 ± 42.76		96.98 ± 39.21	94.61 ± 37.49	77.43 ± 50.94	
C-reactive protein, mean(SD*), mg/dL	0.50 ± 1.80		1.18 ± 3.10	2.67 ± 5.12	5.08 ± 5.78	
**Unfavorable outcome, n(%)**	321(23.0)		43(35.2)	147(43.2)	35(76.1)	<0.001

*Abbreviation: SD, standard deviation; IQR, interquartile range; BMI, body mass index; TIA, transient ischemia attack; mRS, modified rankin scale; NIHSS, National Institutes of Health Stroke Scale; LAA, large artery atherosclerosis; SVO, small vessel occlusion; CE, cardioembolism; LDL, low-density lipoprotein.

**Table 3:** Association between nutritional risk index and 3-month outcomes.

Variables	Unadjusted OR* (95% CI*)	P-value	Adjusted OR* (95% CI*)**	P-value
**Nutritional risk index**				
**No risk**	1 (Reference)		1 (Reference)	
**Mild risk**	1.826 (1.234–2.702)	0.027	1.281 (0.785–2.091)	0.321
**Moderate risk**	2.555 (1.994–3.275)	<0.001	1.522 (1.110–2.086)	0.009
**Severe risk**	10.675 (5.361–21.259)	<0.001	3.838 (1.727–8.529)	0.001
**Age**	1.042 (1.032–1.052)	<0.001	1.034 (1.021–1.046)	<0.001
**Female**	1.660 (1.357–2.030)	<0.001	1.395 (1.086–1.790)	0.009
**Hypertension**	1.343 (1.088–1.658)	0.006	1.344 (1.020–1.772)	0.036
**Diabetes mellitus**	1.446 (1.174–1.781)	0.001	1.374 (1.062–1.778)	0.016
**Dyslipidemia**	0.788 (0.639–0.972)	0.026	0.882 (0.681–1.143)	0.344
**Atrial fibrillation**	2.001 (1.590–2.517)	<0.001	1.182 (0.766–1.822)	0.451
Previous stroke TIA*	1.811 (1.437–2.283)	<0.001	1.417 (1.066–1.883)	0.016
**Stroke subtype**				
LAA*	1 (Reference)		1 (Reference)	
SVO*	1.160 (0.833–1.617)	0.379	1.416 (0.939–2.134)	0.097
CE*	0.722 (0.492–2.425)	0.096	1.317(0.833–2.081)	0.238
**Other determined**	1.736 (1.242–2.425)	0.001	0.778 (0.471–1.286)	0.328
**Undetermined**	2.406 (1.594–3.631)	<0.001	1.034 (1.022–1.047)	<0.001
Premorbid mRS*	1.492 (1.386–1.607)	<0.001	1.264 (1.154–1.384)	<0.001
Initial NIHSS*	1.229 (1.202–1.257)	<0.001	1.217(1.186–1.248)	<0.001

*Abbreviation: OR, odds ratio; CI, confidence interval; TIA, transient ischemia attack; LAA, large artery atherosclerosis; SVO, small vessel occlusion; CE, cardioembolism; mRS, modified rankin scale; NIHSS, National Institutes of Health Stroke Scale

**Adjusted for nutritional risk index, age, sex, history of hypertension, diabetes mellitus, dyslipidemia, atrial fibrillation, previous stroke or transient ischemia attack, stroke subtype, premorbid mRS, and intial NIHSS

## Discussion

In this study, we found that patients with premorbid undernutrition, as screened by the GNRI score, had an unfavorable outcome after acute ischemic stroke. We also found that severe premorbid undernutrition was related to a higher risk of poor outcome in a dose dependent manner, even after adjustment for premorbid state.

Undernourished patients were more likely to have a longer hospitalization duration and a more severe stroke. Undernutrition has a negative effect on brain plasticity associated genes, suppresses protein synthesis and glucose utilisation at the ischemic penumbra, and causes immune suppression which can lead to infection [19–20]. In this context, initial nutritional assessment could be important for prognosis after stroke. In spite of its importance, there is no universally accepted nutritional screening tool, especially for stroke patients. There are tools for screening nutrition status such as malnutrition universal screening tool (MUST) or original nutritional risk index (NRI). However, they need the cooperation of patients to fill out the questionnaires or report their recent weight loss. Additionally, these methods of nutritional assessment have some manner of arbitrary and subjective components, need detailed training of healthcare professionals or normal cognitive function of patient. Therefore, they do not fit for screening all ischemic stroke patients. The biochemical data related to nutritional status, including total cholesterol, serum albumin, transferrin, prealbumin, and CRP, are influenced by medical conditions, including malignancy, liver disease, infection, stress, and critical illness. Therefore, the results of previous studies have been inconsistent in proving the validity of serum markers as determinants of a patient’s nutritional status by themselves [21].

The GNRI is an objective and simple assessment tool, which is a source of competitive strength for a nutritional marker; the GNRI score can be readily calculated automatically in electronic medical record systems. The GNRI has recently been used in elderly patient (over 60 years old), especially with underlying diseases such as heart or kidney problem. Given that most of stroke patients are over 60 years old and have underlying disease, the use of GNRI for stroke patients is worth considering. In another aspect, the GNRI has a high sensitivity for malnutrition compared to other reliable assessment tools [22]. Approximately 20% of the stroke patients had a moderate or severe risk of malnutrition at admission in our study based on the GNRI result, which was within the range reported previously [23].

Serum albumin, also used for nutritional assessment, is largely influenced by extracellular fluid volume status or inflammation [24–27]. For interpretation of the results, CRP level was presented to reduce the confounding effects of inflammation on albumin level in this study. Albeit the lower NRI group had a higher level of CRP, compared to previous studies on inflammation and cardiovascular risk, the level of CRP was lower than that in previous studies even in the lowest GNRI group [28]. We know that albumin level may be influenced by nutritional status and inflammation, but the quantitative relationship between CRP and albumin is still unknown. Therefore we introduced GNRI, the indicator that reflects both body weight and albumin. GNRI has been proposed to use for monitoring the nutritional status of malignancy, perioperative and hemodialysis patients, which is thought to be a disease that can be accompanied by inflammation, just like stroke [8, 29–30]. The use of both weight and albumin indicators in the GNRI minimizes confounding variables such as hydration status and altered albumin level related to comorbidities including inflammation [31].

Although there is limited evidence that nutritional intervention may improve short-term outcomes, recent studies recommend that it is reasonable to be carefully concerned about nutrition [32–33]. These findings could shed the light on screening to malnourished patients in ischemic stroke cases based on the screening results at admission.

There are several limitations to our study. First, the GNRI was assessed only on admission, and was not repeated afterward. Therefore, we do not have information whether GNRI was changed after nutritional support during the hospital stay. This is an important issue but may require further study. Second, we did not assess MUST, so comparison between GNRI and MUST could not be done. Despite these limitations, we think that our data are valid in presenting a correlation between premorbid undernutrition evaluated by the GNRI and functional outcome in patients with ischemic stroke.

## Conclusions

In conclusion, this study demonstrated that GNRI was associated with poor outcomes after ischemic stroke. The GNRI is a simple and sensitive screening tool for malnutrition, allowing quick identification of undernourished stroke patients.

## Supporting information

S1 Table The Database set we used for the study. All relevant data are within the manuscript and its Supporting Information files.
(XLS)
